# Retinoic acid induces white adipose tissue browning by increasing adipose vascularity and inducing beige adipogenesis of PDGFRα^+^ adipose progenitors

**DOI:** 10.1038/celldisc.2017.36

**Published:** 2017-10-10

**Authors:** Bo Wang, Xing Fu, Xingwei Liang, Jeanene M Deavila, Zhixiu Wang, Liang Zhao, Qiyu Tian, Junxing Zhao, Noe Alberto Gomez, Sophie C Trombetta, Mei-Jun Zhu, Min Du

**Affiliations:** 1 Nutrigenomics and Growth Biology Laboratory, Department of Animal Sciences, Washington State University, Pullman, WA, USA; 2 Advanced Innovation Center for Food Nutrition and Human Health, China Agricultural University, Beijing, China; 3 College of Animal Science and Veterinary Medicine, Shanxi Agricultural University, Taigu, Shanxi, China; 4 School of Food Sciences, Washington State University, Pullman, WA, USA

**Keywords:** adipose progenitor, angiogenesis, beige adipogenesis, retinoic acid, vitamin A, VEGF

## Abstract

Formation of beige adipocytes within white adipose tissue enhances energy expenditure, which is a promising strategy to reduce obesity and prevent metabolic symptoms. Vitamin A and its bioactive metabolite, retinoic acid (RA), have regulatory roles in lipid metabolism. Here we report that RA induces white adipose tissue browning via activating vascular endothelial growth factor (VEGF) signaling. RA triggered angiogenesis and elicited *de novo* generation of platelet-derived growth factor receptor α positive (PDGFRα^+^) adipose precursor cells via VEGFA/VEGFR2 signaling. In addition, RA promoted beige/brown adipocyte formation from capillary networks *in vitro*. Using PDGFRα tracking mice, we found that the vascular system acted as an adipogenic repository by containing PDGFRα^+^ progenitors which differentiated into beige adipocytes under RA or VEGF164 treatments. Conditional knockout of VEGF receptors blocked RA-stimulated white adipose tissue browning. Moreover, the VEGFA and RA activated p38MAPK to enhance the binding of RA receptor to RA response elements of the *Prdm16* promoter and upregulated *Prdm16* transcription. In conclusion, RA induces white adipose tissue browning by increasing adipose vascularity and promoting beige adipogenesis of PDGFRα^+^ adipose progenitors.

## Introduction

Due to the global obesity epidemic, there is an urgency for an increased understanding of adipose tissue development and its roles in metabolic dysfunction. While adipocytes are benign and functional at regular sizes, adipocyte hypertrophy can lead to tissue hypoxia [1] and interstitial fibrosis [2], which results in adipose metabolic dysfunction [3]. The formation of brown adipocytes within white adipose tissue (WAT), termed beige adipogenesis, enhances energy expenditure and provides a new alternative for preventing and reducing obesity and other metabolic symptoms [4]. However, contemporary knowledge about the origins of beige adipocytes and mechanisms regulating beige adipogenesis remains rudimentary.

The process of adipogenesis is spatially and temporally associated with vascular development, therefore, high vascularization is observed in mature adipose tissue [5]. The function of this vascularization is not simply limited to providing oxygen and nutrients, but also transporting metabolic products such as transporting growth factors [6] which are critical for adipose tissue expansion and remodeling [7]. Consistently, adipose-derived stem cells of diabetic mice have lower angiogenic potential [8]. It has also been shown that the angiogenic capacity of subcutaneous adipose tissue decreases with morbid obesity [9]. Deletion of vascular endothelial growth factor a (*Vegfa*), a growth factor critical for angiogenesis, reduces vascular density and leads to adipose hypoxia, apoptosis, inflammation and metabolic defects on a high-fat diet [10]. In contrast, *Vegfa* overexpressing increases adipose vasculature and improves insulin sensitivity [10, 11]. It was recently discovered that adipose-specific *Vegfa* overexpression induced WAT browning, which increases energy expenditure [12]. Given that pericytes and endothelial cells can differentiate into adipocytes [13, 14], blood vessels may act as a progenitor pool for adipocyte replenishment [6]. On the other hand, in a three-dimensional culture environment, preadipocytes have the potential to induce endothelial cell migration [15]. Thus, endothelial cells and adipocytes interplay through reciprocal exchange of growth factors, hormones and cytokines [5].

Retinoic acid (RA), a metabolite of vitamin A, is an important regulator of lipid metabolism [16]. Ablation of retinol dehydrogenase 1 (*rdh1*), an enzyme that produces the precursors of RA, in mice leads to adipocyte hypertrophy and adiposity [17]. On the contrary, RA treatment results in weight loss and improves glucose tolerance [18]. In embryonic stem cells, RA promotes adipogenic program at the early stage of adipogenesis, before peroxisome proliferator activated receptor gamma (PPARG) functions [19]. In mature adipocytes, RA activates both retinoic acid receptors (RARs) and peroxisome proliferator activator receptor delta (PPARδ) to enhance lipid oxidation and energy dissipation [18] which reduces lipid accumulation [20]. Correspondingly, RA administration to mice reduces obesity by upregulating uncoupling protein-1 (*Ucp1*) expression both *in vivo* and *in vitro* [16, 21, 22]. However, the role of RA on brown/beige adipogenesis and the underlying mechanisms have yet to be defined. Because RA increases the production of nitric oxide, an important mediator of angiogenesis, in vascular endothelial cells [23] and induces angiogenesis *in vitro* [24], we hypothesized that RA induces angiogenesis which subsequently promotes brown/beige adipogenesis. We previously found that maternal vitamin A supplementation promotes vascular development and increases adipose progenitors [25]. In the current study, we further explored the underlying mechanism by performing both *in vivo* and *in vitro* experiments.

## Results

### RA promotes *in vitro* angiogenesis of stromal vascular cells by stimulating VEGFA/VEGFR2 signaling

**Figure 1:**
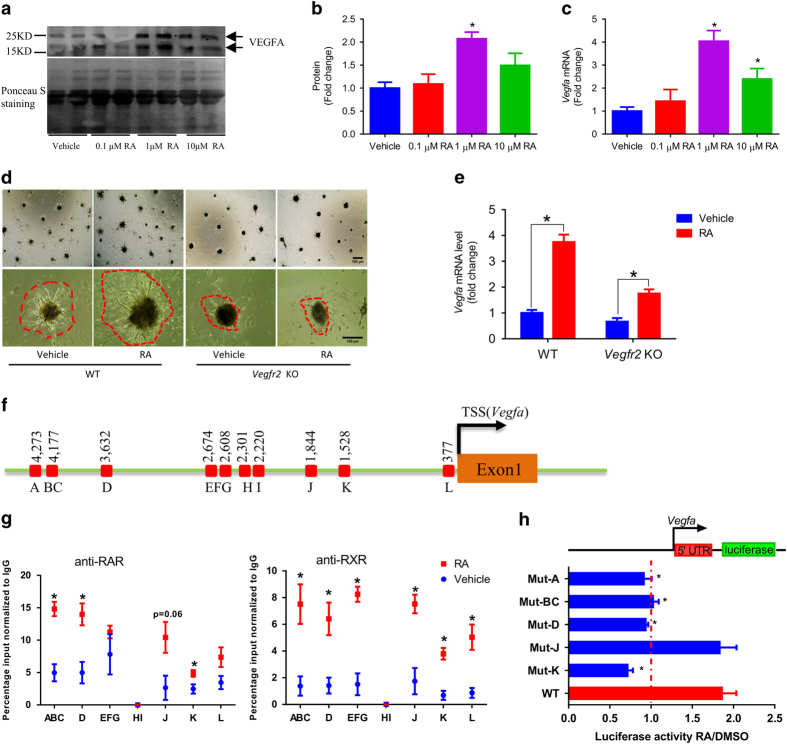
RA promotes *in vitro* angiogenesis of SVCs via VEGFA/VEGFR2 signaling. (**a**) VEGFA protein content in the medium cultured with adipose-derived SVCs for 2 days, and cells were treated with different doses of RA. (**b**) Quantification of VEGFA protein, data presented were normalized according to the content of a non-specific band. (**c**) The expression of *Vegfa* in SVCs treated with RA for 2 days. (**d**) Representative images (scale bar=100 μm), and (**e**) *Vegfa* expression of adipose tissue derived control or *Vegfr2* knockout SVCs cultured on matrigel-coated plates in endothelial basal medium for 2 days with or without 1 μm RA (same doses for other *in vitro* studies if unstated). (**f**) Putative retinoic acid response elements (pRARE) on the *Vegfa* promoter are indicated by letters, some of which share several nucleotides; closely located pRARE were analyzed using one pair of primers. (**g**) SVCs were treated with RA or DMSO for 4 h, and binding of RAR and retinoid X receptor (RXR) to pRAREs were analyzed by ChIP. (**h**) The pRARE sites with altered RAR/RXR binding due to RA were chosen. These sites on a *Vegfa* promoter luciferase reporter plasmid were mutated. The luciferase activity of WT and mutated *Vegfa* promoter reporter plasmids were analyzed after 4 h of RA treatment. Data presented are mean±s.e.m., *n*=6, *P<0.05.

The effects of RA on angiogenesis were tested *in vitro* using adipose tissue stromal vascular cells (SVCs), which contain both preadipocytes and endothelial progenitor cells [26]. Instead of two-dimensional culture system that allows limited cell–cell interactions, three-dimensional culture system were used to provide an appropriate environment for organogenesis *in vitro* [27]. Inguinal WAT (iWAT)-derived SVCs were cultured on matrigel-coated plates in endothelial basal medium supplemented with dimethyl sulfoxide (DMSO) or RA for 2 days. RA-treated cells produced and secreted more VEGFA to the medium (Figure 1a and b) and had higher mRNA level of *Vegfa* (Figure 1c). Because 1 μM RA had the highest effects on both protein (Figure 1a and b) and mRNA levels (Figure 1c), this dose was used in the subsequent *in vitro* experiments. After seeding, SVCs aggregated and formed organoid colonies surrounded by capillary sprouts. The capillary sprouts continuously grew into the Matrigel and free cells migrated to expand the colony. Compared with the control group, RA substantially increased the number of colonies (Figure 1d; Supplementary Figure S1b). When *Vegfr2* was knocked out by CRISPR/Cas9 (Supplementary Figure S1a), the number of cell colonies dramatically decreased (Figure 1d; Supplementary Figure S1b). In addition, RA also increased the number of capillary sprouts and promoted sprout elongation, which was absent in *Vegfr2* null cells even in the presence of RA (Figure 1d; Supplementary Figure S1c and d). Notably, RA upregulated *Vegfa* in both WT and *Vegfr2* null cells (Figure 1e). These data show that RA promotes angiogenesis of the organoids formed from adipose-derived SVCs by activating VEGFA/VEGFR2 signaling.

To address why RA stimulates *Vegfa* expression, we analyzed putative RA response elements (RAREs) in the *Vegfa* promoter and found twelve putative sites (Figure 1f). RA increased the binding of RAR/retinoid X receptor to a number of sites (Figure 1g). We further mutated these sites on the *Vegfa* promoter luciferase reporter plasmid. The mutation of 4 putative RARE sites (pRARE) abolished the activation of *Vegfa* promoter stimulation by RA treatment (Figure 1h), showing that RA directly upregulates *Vegfa* expression via multiple RAREs on its promoter region.

### RA promotes brown adipogenesis *in vitro* through activating VEGFA/VEGFR2 signaling

**Figure 2:**
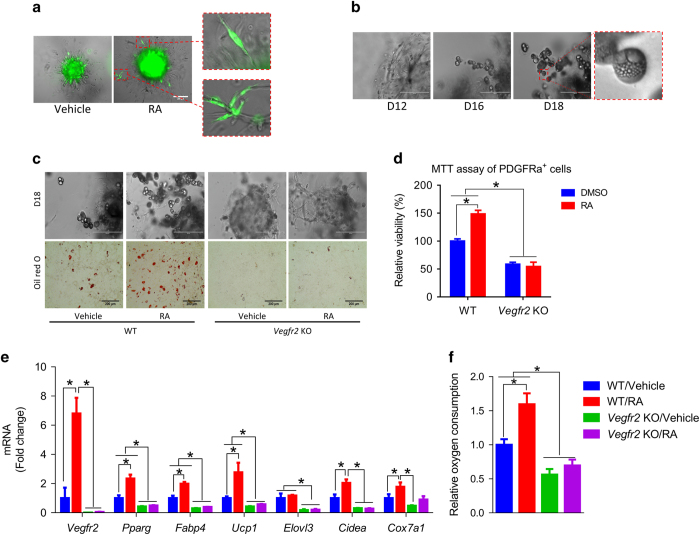
RA promotes brown adipogenesis *in vitro* through activating VEGFA/VEGFR2 signaling. Adipose-derived SVCs were cultured on Matrigel-coated plates in endothelial basal medium medium supplemented with either DMSO or RA for 6 days, then switched into DMEM medium supplemented with insulin and T3 for 4 days, and further cultured with insulin for 8 days. (**a**) PDGFRα^+^ cells at D2. (**b**) Representative images of RA-treated cells at D12, D16 and D18. (**c**) Representative image of D18 control cells and *Vegfr2* knockout cells treated with DMSO or RA (upper: bright field; lower: Oil-Red-O). (**d**) MTT assay of WT or *Vegfr2* KO PDGFRα^+^ cells treated with or without 1 μm RA. (**e**) mRNA levels in D18 cells. (**f**) Oxygen consumption rate of cells. Data presented are mean±s.e.m., *n*=6, *P<0.05. Scale bar=200 μm.

PDGFRα^+^ marks adipose progenitors located in the perivascular region that can differentiate into both brown and white adipocytes. Before adipogenesis, SVC-formed colonies on Matrigel were stained with anti-PDGFRα antibody. More PDGFRα^+^ cells were observed inside RA-treated colonies, and some of them migrated out of vessel boundaries (Figure 2a). After 6 days of angiogenesis on matrigel, to induce brown adipogenesis, cells were switched into DMEM medium supplemented with insulin and triiodothyroxine (T3) for 4 days. Cells were further cultured with insulin for 8 days. The capillary structures continued to extend into the Matrigel. At day 16, adipocytes were found both outside (Figure 2b) and within (Supplementary Figure S2a) capillaries. By day 18, more adipocytes outside the capillaries or colonies were observed (Figure 2b). We observed the loss of continuity between the capillary structure and adipocytes (Supplementary Figure S2b and c), and the presence of adipocyte clusters and free adipocytes suggests adipogenic progenitor proliferation and migration (Supplementary Figure S2b–d). More adipocytes were observed in the RA-treated group, which could be due to increased PDGFRα^+^ adipose progenitors (Figure 2c). Indeed, RA enhanced proliferation of PDGFRα^+^ cells isolated from SVCs (Figure 2d). *Vegfr2* null PDGFRα^+^ cells had lower proliferation rates and did not respond to RA (Figure 2d), suggesting that RA increases PDGFRα^+^ cells by activating VEGFA/VEGFR2 signaling. *Vegfr2* null cells had abnormal capillary development, the capillary sprout rarely grew and very few adipocytes were formed (Figure 2c). After inducing brown adipogenesis, RA upregulated the expression of brown adipose genes including *UCP1*, *Cidea* and *Cox7a1*, which were expressed at much lower levels in *Vegfr2* null cells (Figure 2e). In line with the increased brown adipose genes, RA-treated cells had elevated oxygen consumption rates when compared with control cells (Figure 2f). This suggests that RA-treated cells had a higher metabolic rate, which was not observed in *Vegfr2* null cells (Figure 2f). In summary, these data show that RA, functioning through VEGFA/VEGFR2, promotes angiogenesis, increases PDGFRα^+^ adipose progenitor cells and stimulates the development of beige adipocytes *in vitro*.

### RA activates VEGF signaling to increase PDGFRα^+^ adipose progenitors *in vivo*

**Figure 3:**
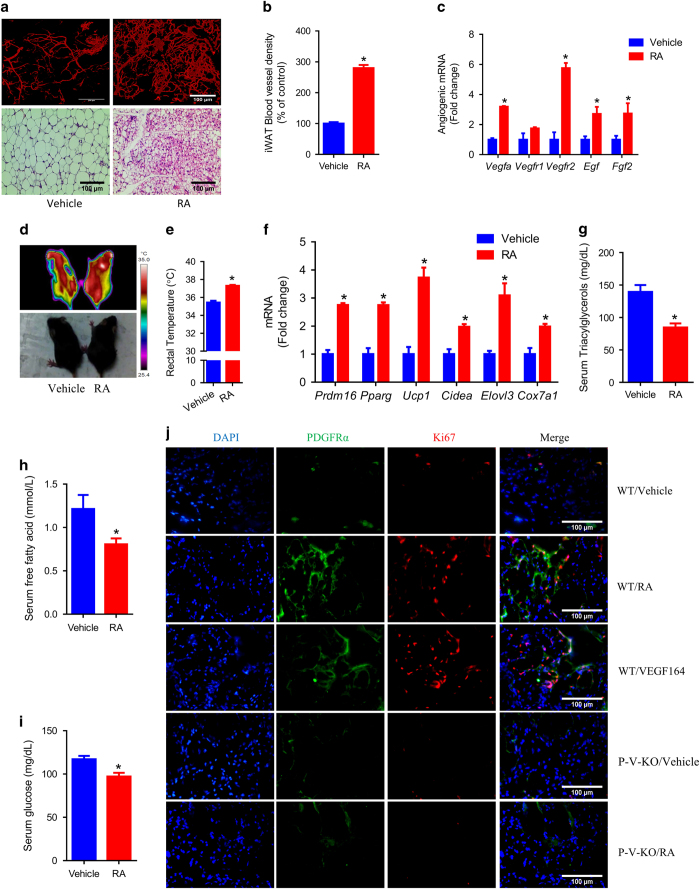
RA activates VEGF signaling to increase PDGFRα+ adipose progenitors *in vivo*. C57BL6 mice were injected with RA (10 mg kg^−1^ BW injected every other day) for 1 week. (**a**) Representative images showing blood vessel and adipocytes in iWAT. (**b**) Quantification of blood vessel density. (**c**) Angiogenic gene expression in iWAT. (**d**) Surface temperature. (**e**) Rectal temperature. (**f**) Brown adipose gene expression in iWAT. (**g**) Serum triacylglycerols. (**h**) Serum-free fatty acids. (**i**) Serum glucose. (**j**) Proliferating (Ki67 labeled) and PDGFRα-positive cells in iWAT of WT and P-V-KO (PDGFRαCreER-VEGFR2^loxP^) conditional VEGFR2 knockout mice in response to 1 week of RA (10 mg kg^−1^ BW) or VEGF164 (2 μg kg^−1^ BW) injection (injected every other day, and same doses for other *in vivo* experiments if unstated). Data presented are mean±s.e.m., *n*=6, *P<0.05. Scale bar=100 μm.

*In vivo*, 1 week RA administration (10 mg kg^−1^ body weight (BW), injected every other day) increased blood vessel density in iWAT, reduced the size of adipocytes and induced the formation of multilocular beige adipocytes in WAT (Figure 3a and b). Consistently, RA upregulated the expression of angiogenic genes including *Vegfa*, *Vegfr1*, *Vegfr2*, epidermal growth factor (*Egf*), fibroblast growth factor 2 (*Fgf2*) in iWAT (Figure 3c) and raised body temperature (Figure 3d and e). RA also upregulated the brown adipose genes in iWAT (Figure 3f). Because of the increased beige adipocytes, RA reduced serum triacylglycerols (Figure 3g), free fatty acids (Figure 3h) and glucose (Figure 3i). As showed by IHC staining, RA and an isoform of the mouse recombinant VEGFA, VEGF164, increased the proliferation of PDGFRα^+^ cells in iWAT of WT (wild type, C57BL6) mice. In PDGFRαCreER-VEGFR2^loxP^ (P-V-KO) mice, in which VEGFR2 was conditionally knocked out in PDGFRα^+^ cells, RA showed no effects on proliferation of PDGFRα^+^ cells (Figure 3j; Supplementary Figure S3). In summary, *in vivo* RA injection induced WAT browning which is accompanied with increased blood vessel density. RA increased adipose progenitors by activating VEGFA/VERGFR2 signaling.

### RA promotes the differentiation of PDGFRα-positive cells into beige adipocytes *in vivo*

**Figure 4:**
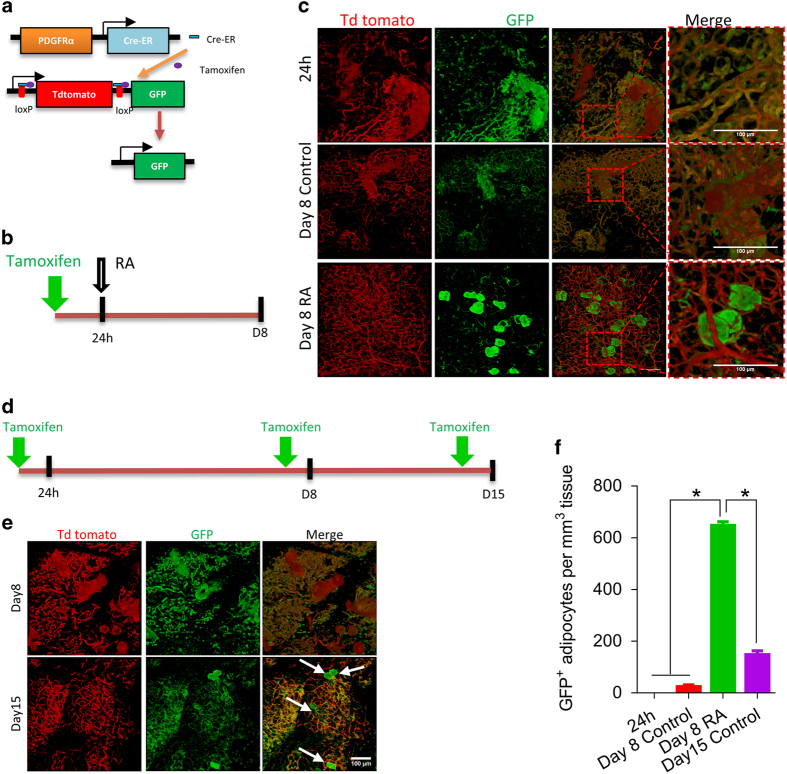
RA promotes the differentiation of PDGFRα-positive cells into beige adipocytes *in vivo*. (**a**) PDGFRα tracking mice. (**b**) Graph showing time points of tamoxifen and RA treatment. (**c**) PDGFRα tracking mice were injected with tamoxifen, then injected with vehicle (DMSO) or 10 mg kg^−1^ BW RA 24 h later. Representative images show blood vessels (red) and PDGFRα^+^ cells (green) in iWAT of PDGFRα tracking mice at different time points. (**d**) Graph showing time points of tamoxifen and RA treatment. (**e**) Representative images of blood vessels and PDGFRα^+^ cells in iWAT of PDGFRα tracking mice injected with tamoxifen once every week. (**f**) Quantification of GFP^+^ adipocytes. Data presented are mean±s.e.m., *n*=6, *P<0.05. Scale bar=100 μm.

To examine the fate of PDGFRα^+^ adipose progenitor cells after RA treatment, we developed PDGFRα tracking mice (Figure 4a) using two transgenic lines: PDGFRαCreER and ROSA^mT/mG^ mice. Without tamoxifen, all cells in PDGFRα tracking mice express tdTomato with red fluorescence. Following tamoxifen (100 mg kg^−1^ BW, 24 h, Figure 4b) administration, the mT cassette was deleted in Cre-expressing cells allowing for GFP expression. GFP positive cells were found on blood vessels and adipose tissue (Figure 4c; Supplementary Videos S1,Supplementary Videos S2, Supplementary Videos S3). Then, mice were treated with RA (Figure 4b), and after 1 week, the amount of PDGFRα^+^ cells decreased and some GFP labeled adipocytes were observed (Figure 4c and f), indicating that the perivascular PDGFRα^+^ cells at least partially differentiated into adipocytes. To determine whether PDGFRα^+^cells would be replenished, PDGFRα lineage tracing mice were further administered with tamoxifen at day 7 (D7) and D14 respectively, so that newly generated PDGFRα^+^ cells would be labeled with GFP (Figure 4d). The population of PDGFRα^+^ cells was constantly present (Figure 4e) despite a portion of PDGFRα^+^ cells that differentiated into adipocytes (Figure 4e and f). These data showed that PDGFRα^+^ cells on blood vessels are capable of self-renewal and a small portion of PDGFRα^+^ cells can differentiate into adipocytes spontaneously. Furthermore, we did whole mount tissue or tissue section immunohistochemical staining using anti-UCP-1 antibody. Those adipocytes derived from PDGFRα^+^ cells were UCP-1 positive (Supplementary Figure S4a and b), showing their beige adipocyte identity. These data suggest that, RA signaling activates the differentiation of PDGFRα^+^ cells, on or around blood vessels, into beige adipocytes.

### VEGF signaling is required for RA-induced WAT browning

**Figure 5:**
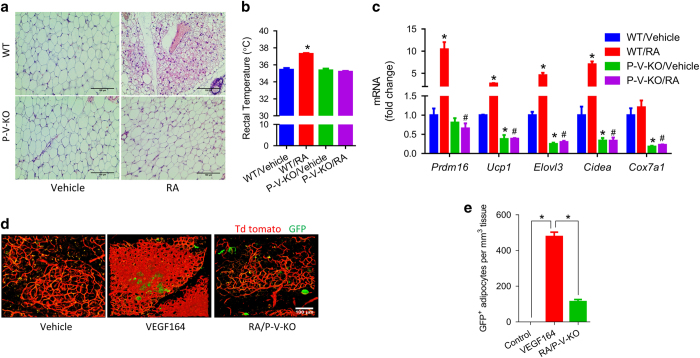
VEGF signaling mediates beige adipogenesis stimulated by RA *in vivo*. (**a**) Representative images of H&E stained iWAT adipocytes in WT and P-V-KO mice injected with vehicle or RA for 1 week, (**b**) rectal temperature, and (**c**) mRNA levels in iWAT of WT and P-V-KO mice treated with RA for 1 week (*compared with WT/Vehicle, ^#^compared with P-V-KO/Vehicle). (**d**) Representative images of iWAT and (**e**) frequency of GFP^+^ cells in PDGFRα tracking mice treated with VEGF164 or RA for 1 week. Data presented are mean±s.e.m., *n*=6, * or ^#^*P*<0.05. Scale bar=100 μm.

VEGF is known to regulate metabolic homeostasis [10], modulate cold tolerance and energy expenditure [28]. Consistent with previous studies, 1 week of VEGF164 injections (2 μg kg^−1^ BW, once per two days) induced iWAT browning (Supplementary Figure S5a) and thermogenic activities (Supplementary Figure S5b and c). VEGF164 upregulated the expression of brown/beige adipose genes *Prdm16*, *Ucp1*, *Elovl3* and *Cox7a1* (Supplementary Figure S5d). Although PDGFRa is expressed in other mesenchymal populations, PDGFRa-Cre mouse line was identified as an efficient model for adipose lineage tracing and can be used for gene ablation at the level of adipose progenitors [29–31]. When *Vegfr2* was knocked out in PDGFRα^+^ cells, RA failed to induce WAT browning (Figure 5a), thermogenesis (Figure 5b) and expression of adipose genes (Figure 5c). Moreover, 1 week of VEGF164 injection (2 μg kg^−1^ BW, once per two days) on PDGFRα tracking mice induced adipogenic differentiation of PDGFRα^+^ cells (Figure 5d and e). Fewer PDGFRα^+^ cells differentiated into adipocytes upon RA injection when *Vegfr2* was conditionally knocked out (Figure 5d and e). These data proved that VEGF signaling in PDGFRα^+^ adipose progenitor cells is required for RA-induced WAT browning.

### RA and VEGF signaling in brown adipogenesis of P19 embryo stem cell line

Embryo bodies (EBs) formed by P19 embryonal carcinoma stem cells have the capacity to differentiate into three germ layer cells [32], and have been used for studying early differentiation of adipocytes [19] and endothelial cells [33], and morphogenesis of adipose tissue [19]. RA-treated EBs had higher expression of *Vegfa*, *Vegfr1* and *Vegfr2*, which was downregulated in BMS493, a pan-retinoic acid receptor inverse agonist, treated EBs (Supplementary Figure S6a). Moreover, more VEGFR2 positive cells (Supplementary Figure S6b) and increased cell expansion (Supplementary Figure S6c) were found in the RA-treated group. Using P19 cells, we explored the interaction between RA and VEGF signaling on early brown adipogenic differentiation.

**Figure 6:**
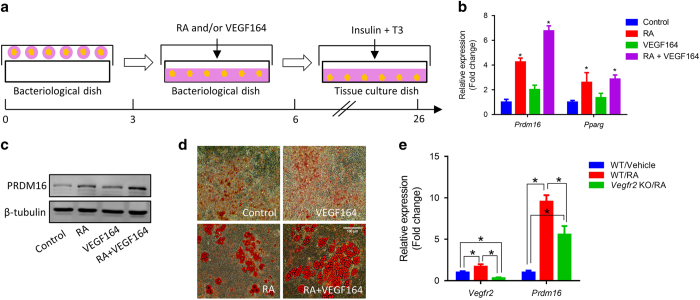
RA and VEGF signaling in brown adipogenesis of P19 embryo stem cell line. (**a**) P19 embryo bodies were formed in hanging drops then treated with VEGF164 (10 nm, same dose for other *in vitro* experiments if unstated) or RA for 3 days, then underwent brown adipogenesis for 26 days. (**b**) *Prdm16*, *Pparg* mRNA and (**c**) PRDM16 protein after treated with RA or VEGF164 for 3 days. (**d**) P19 cell-derived adipocytes stained by Oil-Red O. Scale bar=100 μm. (**e**) The expression of *Vegfr2* and *Prdm16* in WT and *Vegfr2* KO p19 cells in the presence or absence of RA during brown adipogenesis. Data presented are mean±s.e.m., *n*=3, *P<0.05.

We treated P19 embryo bodies with RA and/or 10 nm VEGF164, as well as DMSO (control) for 3 days (Figure 6a). Brown adipogenesis was then induced using insulin and T3. After 4 days, RA dramatically increased *Prdm16* and *Pparg* expression (Figure 6b). When combined with RA, VEGF164 showed synergistic effects on the expression of brown adipogenic genes (Figure 6b). Similarly, RA increased PRDM16 protein level and VEGF164 showed similar but much weaker effects on PRDM16 (Figure 6c; Supplementary Figure S6d). Although VEGF164 upregulated brown adipogenic genes, only small lipid droplets were accumulated in VEGF164-treated cells which showed no difference to the control cells (Figure 6d). Embryo bodies pretreated with RA differentiated into adipocytes with dramatically larger lipid droplets (Figure 6d). We further identified the P19 cells-derived adipocytes as brown adipocytes (Supplementary Figure S6e). These data suggest that RA committed the pluripotent cells into brown adipocyte precursors. Using CRISPR/Cas9 system, we knocked out VEGFR2 in P19 cells. The effects of RA on *Prdm16* expression was partially abolished by VEGFR2 ablation (Figure 6e). VEGFA, together with RA, upregulates *Prdm16* expression. However, VEGFa alone is insufficient to induce the adipogenic program of stem cells.

### RA and VEGF activate p38MAPK to recruit RAR to the prdm16 promoter

**Figure 7:**
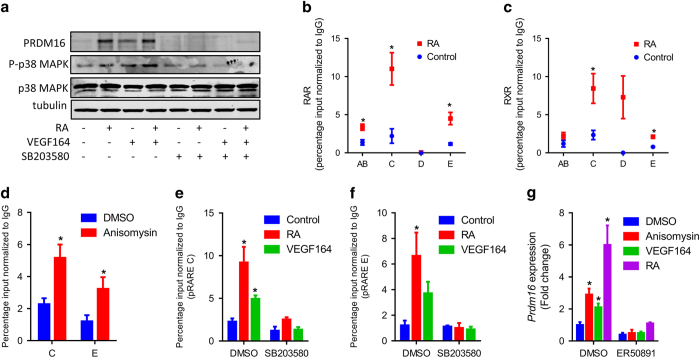
RA and VEGF activate p38MAPK to recruit retinoic acid receptor (RAR) to the *Prdm16* promoter. (**a**) PRDM16, phospho-p38MAPK and p38MAPK proteins in P19 embryo bodies treated with indicated chemicals for 3 days. (**b**, **c**) P19 embryo bodies were treated with RA or DMSO for 4 h, and binding of RAR and retinoid X receptor (RXR) to putative RAR response elements (pRAREs) were analyzed by ChIP. (**d**) Binding of RAR to pRARE #C and #E in embryo bodies (EBs) treated with DMSO or anisomysin for 4 h. (**e**, **f**) Binding of RAR to pRARE #C and #E in EBs treated with DMSO, SB203580, RA or VEGF164 for 4 h. (**g**) Expression of *Prdm16* in EBs treated with indicated chemicals for 4 h. Data presented are mean±s.e.m., *n*=3, *P<0.05.

We further explored whether RA and VEGF164 promote *Prdm16* expression through activation of p38MAPK. P19 EBs were treated with RA or VEGF164 in presence or absence of 10 μM SB203580, the p38MAPK inhibitor. Both RA and VEGF164 increased PRDM16 and phosphorylated p38MAPK protein levels (Figure 7a). SB203580 completely abolished p38MAPK activation and PRDM16 expression (Figure 7a), showing that p38MAPK mediates *Prdm16* expression stimulated by RA.

P38MAPK phosphorylates RARα and directs it to target promoters [34]. Here we identified two RARE sites on *Prdm16* promoter (pRARE C and pRARE E, Figure 7b and c; Supplementary Figure S7b) and confirmed that activation of p38MAPK is related to phosphorylation of RAR (Supplementary Figure S7a). P38MAPK activation by anisomysin, RA or VEGF164 increased the binding of RAR to RAREs on the *Prdm16* promoter whereas p38MAPK inhibition by SB203580 eliminated the effects of RA or VEGF164 on RAR binding (Figure 7d–f). Moreover, in the presence of an RARα selective antagonist, ER50891 [35], anisomysin, VEGF164 or RA failed to upregulate *Prdm16* expression (Figure 7g).

## Discussion

RA signaling acted as a central regulator of adipose tissue remodeling and adipocyte differentiation that is required for the process of WAT browning. (1) RA upregulated the expression of VEGFA which further increased the number of blood vessels in WAT. (2) RA and VEGF induced beige adipogenesis of PDGFRα^+^ adipose precursor cells. (3) RA upregulated the transcription of *Prdm16* to trigger the commitment of pluripotent stem cells into brown adipocyte precursors. Both RA and VEGF signaling activated p38MAPK and further enhanced the binding of RAR to RAREs on the promoter of *Prdm16*, driving brown/beige adipogenesis of progenitor cells.

As an endothelial growth factor, VEGF is well known to induce cell proliferation [36]. In this study, we found RA signaling activates downstream VEGF-VEGFR2 to promote proliferation of PDGFRα^+^ progenitors. Blood vessel walls may be integral to the origin of mesenchymal stem cells and other adult stem cells because of the pluripotency of perivascular cells which is similar to that of mesenchymal stem cells [13]. Besides pericytes, endothelial cells which are traced by VE-cadherin give rise to both white and brown adipocytes [14]. Other adipose progenitor cells like PDGFRα^+^ [37] and PDGFRβ^+^ cells are also perivascularly located. Besides enhanced cell proliferation, the increased angiogenesis under RA treatment would also contribute to the increase of adipose progenitors.

**Figure 8:**
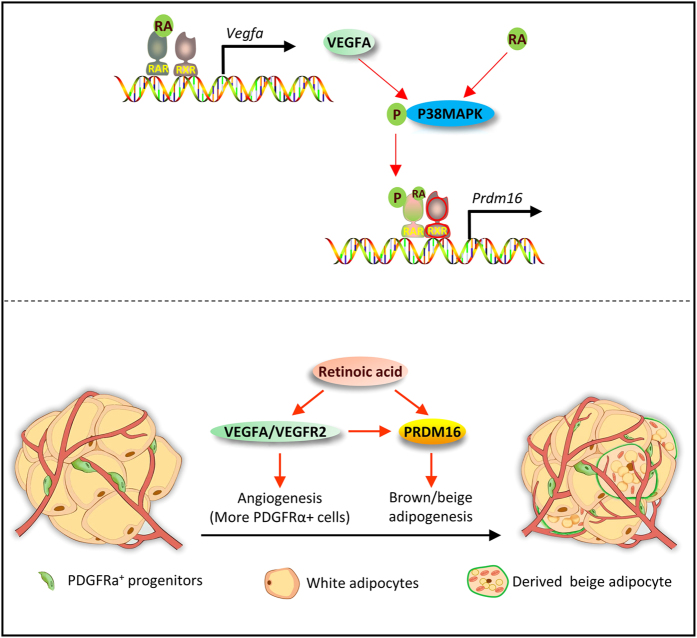
Schematic illustration showing the mechanism of RA-induced WAT browning. Upper: RA binds to RAR/retinoid X receptor heterodimer to activate *Vegfa* promoter. VEGFA and RA activate P38MAPK, and P38MAPK phosphorylates RAR and directs it to the *Prdm16* promoter. Lower: RA activates VEGFA/VEGFR2 signaling to stimulate angiogenesis and increases PDGFRα^+^ cells; RA and VEGFA/VEGFR2 signaling upregulates *Prdm16* to induce white adipose tissue browning.

Angiogenesis is associated with adipose development [7]. However, controversy exists regarding the role of angiogenesis in obesity development. Angiogenesis inhibitors reduce body weight [38, 39] while VEGF overexpression prevents adiposity [10]. Enhanced angiogenesis increases energy expenditure in the metabolically active adipose tissue, while in the metabolically quiescent obese individuals with a large amount of WAT, it is therapeutically beneficial to inhibit angiogenesis [7]. Our data showed that VEGFA promotes the proliferation of PDGFRα^+^ precursor cells, although VEGFA had synergistic effects on *Prdm16* expression by activating p38MAPK, itself is unable to activate *Prdm16* transcription which relies on binding of RA to RAR. The activated VEGFA signaling and increased adipose vascularity provide cells which can differentiate into beige (anti-obesity) or white (pro-obesity), and RA is important in driving these cells to differentiate into beige adipocytes to prevent obesity (Figure 8).

## Materials and methods

### Antibodies and chemicals

Antibodies against β-tubulin (#2146), p38 (#9212), Phospho-p38 (#4511), p38 (#9212) were purchased from Cell Signaling (Danvers, MA, USA), UCP1 (cat. no. PA1-24894), PRDM16 (cat. no. PA5-20872) and VEGFA (cat. no. PA1-16948) from Thermo Fisher Scientific (Waltham, MA, USA). PDGFRα (cat. no. 1062-PR) from R&D. Alexa Fluor 488 anti-mouse CD309 (cat. no. 136408), APC anti-mouse CD140a (cat. no. 135908), PE/Cy7 anti-mouse CD45 (cat. no. 103114) from Biolegend (San Diego, CA, USA). Antibody against ZFP423 (sc-48785) was purchased from Santa Cruz Biotech (Dallas, TX, USA).

1,1′-Dioctadecyl-3,3,3′,3′-tetramethylindocarbocyanine perchlorate (42364), tamoxifen (T5648), all-trans-retinoic acid (R2625), 4-hydroxytamoxifen (H7904), insulin (I3536), dexamethasone (D4902), 3-isobutyl-1-methylxanthine (I5878), triiodothyronine (T3) (IRMM469) and Oil-Red O (O0625) were purchased from Sigma (St Louis, MO, USA). BMS493 (cat. no. 3509) was purchased from Tocris Bioscience (Ellisville, MO, USA). Mouse recombinant VEGF164 (cat. no. 583106) was purchased from Biolegend (San Diego, CA, USA).

### Adipose-derived stromal vascular cell isolation, cell culture and induction of adipogenesis

Stromal vascular cells were isolated from iWAT as previously described [25]. Cells were seeded on 24-well culture dishes pre-coated with 200 μl of Matrigel (8 mg ml^−1^) and covered with 500 μl endothelial basal medium supplemented with 10% FBS for 6 days. Brown adipogenesis was then induced by 10%FBS/DMEM supplemented with 2 μg ml^−1^ insulin and 2 nm T3 for 6 days. Differentiated cells were fixed in 4% PFA for 10 min at room temperature and rinsed 3 times with PBS. Fixed cells were stained with Oil-Red O solution for 10 min then rinsed with PBS to remove excessive Oil-Red O dye. Following microscopic observation, Oil-Red O dye retained in cells were solubilized with isopropanol and the light absorbance was measured at 510 nm using a Synergy H1 Multi-Mode Reader (BioTek, Winooski, VT, USA). For gene expression, before mRNA and protein extraction, cells were incubated with 0.5 U ml^−1^ Dispase in the medium for 30 min at 37 °C to dissolve the Matrigel.

PDGFRα^+^ cells were isolated from stromal vascular cells of inguinal WAT using a manual magnetic cell separation system (Miltenyi Biotec, Bergisch Gladbach, Germany). Isolated PDGFRα^+^ cells were cultured on a 10 cm dish for 24 h, then seeded into 96-well plates (2 000 cells per well) and cultured for 24 h. For analyzing cell proliferation, cells were incubated with sterile 3-(4,5-dimethythiazol-2-yl)-2,5-diphenyltetrazoliumbromide (MTT, Sigma, 0.5 mg ml^−1^ in medium) for 4 h at 37 °C. The culture medium was removed afterwards and 100 μl of DMSO was added to each well. The mixture was further incubated at 37 °C for 10 min and the absorbance was measured at 570 nm using the Synergy H1 Multi-Mode Reader (BioTek, Winooski, VT, USA).

Proteins in cell culture medium were precipitated using chloroform and methanol. Briefly, 1 volume (*V*) of methanol and ¼*V* of chloroform was added to the medium, mixed and centrifuged at 12 000×*g* for 10 min. The methanol layer was discarded (upper layer) and then 1*V* of methanol was added and centrifuged at 12 000×*g* for 10 min. The supernatant was then discarded and the resulting pellet was air dried for subsequent western blot analysis.

### Adipogenesis of P19 cells

Hanging drops containing 10^3^ P19 cells in 25 μl medium were maintained for 3 days on the lids of bacteriological dishes. The embryoid bodies (EB) formed were then transferred into bacteriological dishes in suspension in a medium supplemented with DMSO, all-*trans* RA or VEGF164 for 3 days [19]. EBs were then transferred to tissue culture plates along with differentiation medium supplemented with 2 μg ml^−1^ insulin and 2 nm triiodothyroxine (T3) for 20 days.

### Oxygen consumption assay

Oxygen consumption was measured using a Thermo Scientific Orion 3-Star Dissolved Oxygen Meter (Thermo Electron Corporation, Madison, WI, USA). Fresh culture medium was added to the plates, and the dissolved oxygen concentration was measured at the start and after 30 min incubation.

### CRIPSR/Cas9 gene editing

CRIPSR/Cas9 plasmid expressing a gRNA targeting *Vegfr2* (CRISPR sequence: GTCCCGGTACGAGCACTTGT, Vector: PX458 [40]) and scrambled sequence (CRISPR sequence: GCACTACCAGAGCTAACTCA, Vector: PX458) was generated by GenScript Inc (Piscataway, NJ, USA). All plasmids used in this study were delivered to cells using a Lipofectamin 3000 Reagent (cat. no. L3000015; Thermo Fisher Scientific).

### Bioinformatics and chromatin immunoprecipitation assay

The ChIP assay was performed as previously described [41]. Briefly, the protein-DNA complexes were crosslinked by incubating the cells with 1% formaldehyde solution for 10 min at room temperature (RT). Then, the formaldehyde was quenched by 125 mm glycine for 5 min at RT. Cells were then lysed in a cold lysis buffer (1% SDS, 10 mmol l^−1^ Tris-HCl, pH 8.0, 10 mmol l^−1^ NaCl, 3 mmol l^−1^ MgCl_2_ and 0.5% NP-40) containing a protease inhibitor cocktail (Thermo Fisher Scientific). The samples were then sonicated to shear chromatin to an average length of about 1 kb. After centrifugation at 12 000×*g* for 10 min, the supernatant was pre-cleaned with protein A beads (Thermo Fisher Scientific) and then incubated with antibodies against RAR, retinoid X receptor, or normal rabbit IgG overnight at 4 °C. Then, the antibody-chromatin complex was precipitated with protein A beads, further treated with RNaseA and then proteinase K for 2 h to remove RNA and protein respectively. DNA was purified with ChIP DNA Clean & Concentrator (Zymo Research). The putative RAREs in the *Vegfa* and *Prdm16* promoters were predicted (Supplementary Tables S2 and S5) using the JASPAR database (http://jaspardev.genereg.net/). Primers covering these sites are listed in Supplementary Tables S3 and S6.

### Plasmid DNA mutation and luciferase activity assay

The wild type *Vegf*-luc plasmid (#27987) was purchased from Addgene (Cambridge, MA, USA). *Vegfa* promoter mutations were performed using a Q5 Site-Directed Mutagenesis Kit (NEB, Ipswich, MA, USA). The mutagenic primers were designed using the NEB online primer design software NEBaseChanger (http://nebasechanger.neb.com), these primers are listed in Supplementary Table S4. Luciferase activity was measured using the Dual-Luciferase Reporter Assay System (Promega, cat. no. E1910).

### Mice

Animal studies were conducted in AAALAC-approved facilities according to protocols approved by the Institutional Animal Care and Use Committee (IACUC) at Washington State University. C57BL/6 mice, PDGFRαCreER (stock number: 018280), VEGFR2^loxP^ (stock number: 018977), and R26CreER mice (stock number 004847) and ROSA^mT/mG^ mice (stock number: 007676) mice were purchased from the Jackson Laboratory (Bar Harbor, ME). R26-RARα403 mice were kindly provided by Dr Cathy Mendelsohn [42]. All mice were fed with a diet containing 15 IU g^−1^ Vitamin A (Teklad global diet 2018).

Retinoic acid (10 mg kg^−1^ BW), BMS493 (10 mg kg^−1^ BW) or vehicle (DMSO) dissolved in corn oil were intraperitoneally injected once per two days for mouse experiments. VEGF164 was dissolved in phosphate-buffered saline (PBS) at a dose of 2 μg kg^−1^ BW per injection [43]. PDGFRα tracking mice were given a single intraperitoneal injection of 100 mg kg^−1^ BW tamoxifen to label PDGFRα^+^ cells. Conditional knockouts were induced by daily intraperitoneal injection of 25 mg kg^−1^ BW tamoxifen for three days. Mice lacking Cre or lox genes were used as the WT control for transgenic mice; both control and Cre-lox mice were injected with tamoxifen. The doses of chemicals were determined by our preliminary experiments.

### Intraperitoneal glucose tolerance test

Following 6 h-fasting, mice were administered 2 g kg^−1^ BW d-glucose. Blood samples were collected from the tail veil at 0, 15, 30, 60, 90 and 120 min post injection and glucose concentration was measured using a glucose meter (Bayer Contour, Tarrytown, NY, USA). The area under the curve was calculated.

### Tissue processing and histology

Blood vessels were labeled with 1,1′-dioctadecyl-3,3,3′,3′-tetramethylindocarbocyanine perchlorate (DiI) [44]. Briefly, mice were euthanized by CO_2_, then sequentially perfused with PBS, DiI and 4% PFA into left ventricle (open the right atrium before perfusion). Tissues were then sectioned for further whole mount staining or imaging under a Leica TCS SP8 confocal microscope (Wetzlar, Germany). Blood vessel density was quantified by Image J (NIH). For whole mount staining, tissues were blocked with 5% goat serum in TBS containing 1% Triton X-100 and 0.2% sodium azide for 2 h, then incubated sequentially with primary antibodies for 4 days and secondary antibodies for 2 days.

The PDGFRα tracking mice were perfused with PBS and 4% PFA after they were euthanized by CO_2_. Tissues were then sectioned for direct examination and imaging under a Leica TCS SP8 confocal microscope or further whole mount staining. The confocal microscope images were processed to create 3D videos using Fiji Imaging Processing Package [45]. The PDGFRα derived cells in random fields of tissue sections for each animal were counted and the average per animal was used for quantification.

Other histochemical and immunostaining of adipose tissues were conducted as previously described [46]. Briefly, adipose tissues were fixed in 4% PFA for 12 h at 4 °C, then paraffin embedded and sectioned. Following deparaffinization, tissue sections were used for H&E or immunostaining. For immunostaining, sections were heated in citrate buffer for 20 min, blocked with 5% goat serum in TBS containing 0.3% Triton X-100 for 2 h, then incubated sequentially with primary antibodies overnight and secondary antibodies for 1 h. Sections were then mounted on a mounting medium (Vector Lab, Burlingame, CA, USA).

### Body temperature

Rectal temperatures were measured using a TH-5 Thermalert Monitoring Thermometer (Physitemp Instruments, Inc., Clifton, NJ, USA). Body surface temperatures were measured using a FLIR E6 thermal imaging camera (FLIR System, Wilsonville, OR, USA).

### Real-time quantitative PCR

qRT-PCR was performed as previously described [47]. Primers are listed in Supplementary Table S1.

### Serum profile analysis

Glucose concentration was measured using a glucose meter (Bayer Contour, Tarrytown, NY, USA). Serum triglyceride was analyzed using a triglyceride colorimetric assay kit purchased from Cayman (Ann Arbor, MI, USA, cat. no. 10010303). Serum-free fatty acids level was analyzed using an EnzyChrom Free Fatty Acid Assay Kit (BioAssay Systems, Hayward, CA, USA, cat. no. 50489265).

### Immunoblotting analysis

Proteins were extracted from tissue or cells using a lysis buffer (1% SDS, 10 mmol l^−1^ Tris-HCl, pH 8.0, 10 mmol l^−1^ NaCl, 3 mmol l^−1^ MgCl_2_, 0.5% NP-40, and 10 mmol l^−1^ NaF). Western blotting was performed as previously described [47]. Protein bands were visualized by the Odyssey Infrared Imaging System (LI-COR Biosciences, Lincoln, NE, USA).

### Statistical analysis

Previous experiments done were used to determine sample size with adequate statistical power. Results were analyzed using unpaired, 2-tailed Student’s *t*-test or one-way ANOVA (for multiple comparison) where appropriate, using SAS 9.0 (SAS Institute Inc., Cary, NC, USA). All data were found to be normally distributed. Significance was accepted at *P*<0.05. All data are reported as mean±s.e.m.
