# Supplementary material for: Retinoic acid induces white adipose tissue browning by increasing adipose vascularity and inducing beige adipogenesis of PDGFRα+ adipose progenitors
Source: Cell Discov. 2017 Oct 10;3:17036–. doi: 10.1038/celldisc.2017.36 (PMC5633810; doi:10.1038/celldisc.2017.36)
Supplement: Supplementary Information [file celldisc201736-s1.pdf]

Retinoic acid induces white adipose tissue browning by increasing adipose vascularity and inducing beige adipogenesis of PDGFRα^{+} adipose progenitors

Bo Wang[1,2], Xing Fu[1], Xingwei Liang[1], Jeanene M. Deavila[1], Zhixiu Wang[1], Liang Zhao[1], Qiyu Tian[1], Junxing Zhao[4], Noe Alberto Gomez[1], Sophie C. Trombetta[1], Mei-Jun Zhu[3], and Min Du[1,2*]

[1]Nutrigenomics and Growth Biology Laboratory, Department of Animal Sciences, Washington State University, Pullman, WA 99164, USA;

[2]Advanced Innovation Center for Food Nutrition and Human Health, China Agricultural University, Beijing 100193, China;

[3]School of Food Sciences, Washington State University, Pullman, WA 99164, USA.

[4]College of Animal Science and Veterinary Medicine, Shanxi Agricultural University, Taigu, Shanxi, 030801, China.

[*]Address correspondence to:

Min Du, Ph.D.
Integrative Physiology and Neuroscience,
Washington State University, Pullman, WA 99164;
Tel: 509-335-2744; Fax: 307-766-2355;
E-mail: min.du@wsu.edu

Supplementary Table S1. The primer sequences used for real-time quantitative PCR

Gene name	Forward	Reverse	Product size (bp)
Prdm16	CAGCACGGTGAAGCCATTC	GCGTGCATCCGCTTGTG	87
Ucp1	ACTGCCACACCTCCAGTCATT	CTTTGCCTCACTCAGGATTGG	123
Vegfa	TGGACCCTGGCTTTACTGCT	GCAGTAGCTTCGCTGGTAGA	126
Vegfr1	GGCCCGGGATATTTATAAGAAC	CCATCCATTTTAGGGGAAGTC	71
Vegfr2	CAGTGGGATGGTCCTTGCAT	ACGGTGGTGTCTGTGTCATC	177
Egf	GTTAGCACCATCCCTCATCC	TCTGAGTGCAGCCGGAAAG	246
Fgf2	GGCTGCTGGCTTCTAAGTGT	GTCCCGTTTTGGATCCGAGT	153
Cidea	ATCACAACTGGCCTGGTTACG	TACTACCCGGTGTCCATTTCT	136
Elovl3	GATGGTTCTGGGCACCATCTT	CGTTGTTGTGTGGCATCCTT	73
Cox7a1	CAGCGTCATGGTCAGTCTGT	AGAAAACCGTGTGGCAGAGA	112
18s	TTGTACACACCGCCCGTCGC	CTTCTCAGCGCTCCGCCAGG	102

Supplementary Table S2. Computational analysis of putative RARE sites on the *Vegfa* promoter

pRARE ID	Model ID	Model name	Strand	Putative RARE sites
A	PB0053.1	Rara_1	-1	TACCAAATGTCACGTC
B	PB0053.1	Rara_1	1	TGACCAAGGTCACACT
C	MA0159.1	RXR:RAR_DR5	-1	TGGTCAGCACCAGTTCA
D	MA0159.1	RXR:RAR_DR5	-1	AGGTGAGCAAAAGGCTA
E	PB0053.1	Rara_1	-1	GAGGAAGGGTCACTTC
F	PB0157.1	Rara_2	-1	GAAGCTGGGTCAGAGG
G	PB0157.1	Rara_2	1	TGATCCGGGTTACTCC
H	PB0053.1	Rara_1	-1	TGGGAAAGGCCACGGG
I	PB0157.1	Rara_2	-1	CTGGGGGGGTCACCAC
J	MA0159.1	RXR:RAR_DR5	1	GGATCAGGATCAGGTGA
K	PB0157.1	Rara_2	1	AGATCTGGGTGAGGGC
L	PB0053.1	Rara_1	1	AGCCAGGGGTCACTCT
	PB0157.1	Rara_2	1	AGCCAGGGGTCACTCT

Supplementary Table S3. The primer sequences used for ChIP-PCR targeting *Zfp423*

pRARE ID		Primer sequences
ABC	F	GGTATCAAGGAACAGTCTCAGC
	R	CATCGTTTGCCCTAAGCAGC
D	F	CCTGAAACTCCTGCTTCTCCC
	R	ATGGGGCACAGACTCCCTA
EFG	F	GCTCCAAACCTCAGAATCCTTTACT
	R	CTCAACCCTTTGGAGGGACT
HI	F	GCTACATTTGGTGTTCTTGCGGT
	R	GCTATCCCTTCCTGCCCATCATTAC
J	F	TACTTGTCACTTCCCCTCTTCTTTC
	R	ATTTCCCTCTCCAAAGCACTAAAGC
K	F	CAGAGGGATGGAGATAGTGGATTAC
	R	CTAACCCACCCTTGGATATAGGAAC
L	F	ATCGTGAACTTGGGCGAGC
	R	AGAGTATTGGGAATCAGGGGAGA

Supplementary Table S4. Primer sequences for mutagenesis on the *Vegfa* promoter-luciferase reporter plasmid

pRARE ID	Wild type sequences	Mutated sequences	Mutation primer sequences (forward)	Mutation primer sequences (reverse)
A	taccaaatgtcacgtc	gacgactttgacggta	tgacggtaTCAAGGAACAGTCTCAGCTGTC	aagtcgtcTTCGGGTGGCCTGTGTCC
BC	tgaactggtgctgaccaaggtcacact	gtgataggtgcgagctacaaagtcact	ctacaaagtcactGCCTGTGGCCTTCTCAGC	ctcgcacctatcacCCAGTCCTGCTGCTGGCC
D	tagccttttgctcacct	gagtgttttgcgcgcat	gcgcgcatGCAGACGGGAAGTCTCTTC	aaaacactcAAGTGATGGGCTACATAACTG
J	ggatcaggatcaggtga	gacctaggatttaacac	tttaacacGGAAACCCACAGGAGGGC	tcctaggtcCTGCTCTAAGCAACTAAGAGGAG
K	agatctgggtgagggc	agataatccgacgggc	cgacgggcTGGGACTGGTTGGTCCCT	gattatctGCTGGACTTCTGAGAAAGAGAAC

Supplementary Table S5. Computational analysis of putative RARE sites on the *Prdm16* promoter

pRARE ID	Model ID	Model name	Strand	Putative RARE sites
A	PB0053.1	Rara_1	1	tctcaagggacacctg
B	PB0053.1	Rara_1	-1	ggctcaaggtcaccta
C	MA0159.1	Rara_2	-1	caggctgggtcactgc
D	MA0159.1	Rara_2	1	ggagccgggttagggc
E	MA0159.1	Rara_2	-1	atagttgggacacggg

Supplementary Table S6. The primer sequences used for ChIP-PCR targeting *Prdm16*

pRARE ID		Primer sequences
AB	F	ATCAGGGTCCCTCGTTAGTAG
	R	GCTACACGGGAGATTCCGC
C	F	GTTGCCTTAGAGGGACCTTGG
	R	CAGGGTGGCCCCAACTAAGA
D	F	TCGGCTGTTTGTTTTTGAGCCG
	R	GCTGCTTTTGCTGCGTTTTATTG
E	F	ACGGCGAACGAAAGCTTTTTA
	R	AAACGCGCTGATAGTTGGGA

Supplementary Figures

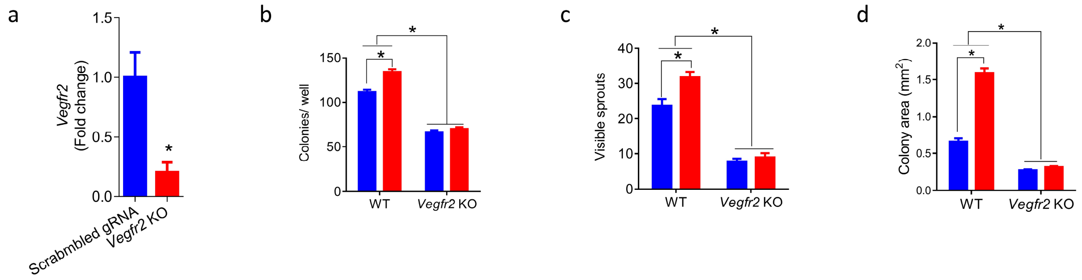

Supplementary Figure S1. RA promotes *in vitro* angiogenesis of SVCs via VEGFA/VEGFR2 signaling. (a) *Vegfr2* expression, (b) Colony number, (c) Visible sprouts, and (d) Colony area of adipose tissue derived control or *Vegfr2* knock out SVCs cultured on matrigel coated plates in endothelial basal medium (EBM) for 2 days. Data presented are mean ± SEM, n = 6, **p* < 0.05.

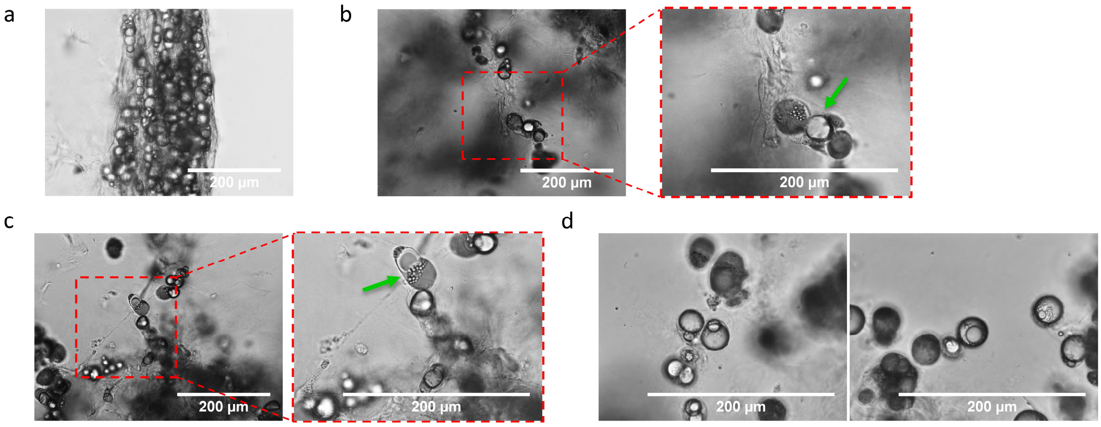

Supplementary Figure S2. RA promotes brown adipogenesis *in vitro* through activating VEGFA/VEGFR2 signaling. Adipose tissue derived stromal vascular cells (SVCs) were cultured on matrigel coated plates in EBM medium supplemented with RA for 6 days, switched into DMEM medium supplemented with insulin and T3 for 4 days, then with insulin for 8 days. (a) Representative image showing adipocytes within a capillary vessel. (b-c) Representative image showing adipocytes within and beside capillaries. Green arrow indicates cells that were leaving the capillary vessel. (d) Representative images of free adipocytes. Scale bar = 200 µm.

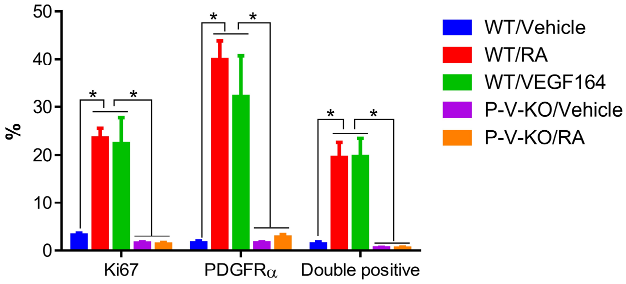

Supplementary Figure S3. Quantification of proliferating (Ki67 labeled) and PDGFRα positive cells in iWAT of WT and P-V-KO (PDGFRαCreER-VEGFR2^{loxP}) conditional VEGFR2 knock out mice in response to RA or VEGF164 injection. Data presented are mean ± SEM, n = 6, **p* < 0.05.

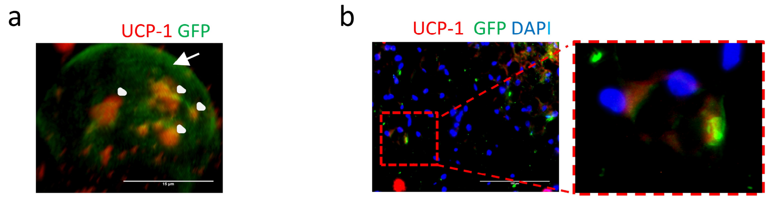

Supplementary Figure S4. RA promotes the differentiation of PDGFRα positive cells into beige adipocytes *in vivo*. UCP1 (arrowhead indicated) in PDGFRa (arrow indicated) derived adipocytes was observed by whole mount tissue (a, scale bar = 15 μm) or tissue section (b, scale bar = 100 μm) IHC staining.

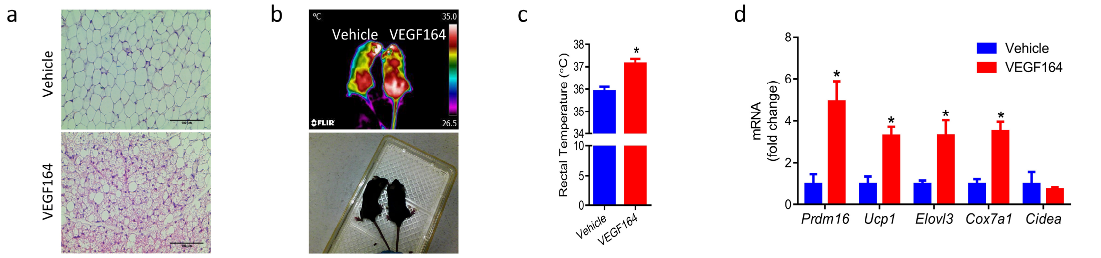

Supplementary Figure S5. VEGF signaling mediates beige adipogenesis stimulated by RA *in vivo*. Mice treated with VEGF164 for 1 week. (a) Representative images of iWAT. (b) Thermal images. (c) Rectal temperature. (d) Gene expression in iWAT. Data presented are mean ± SEM, n = 6, *$p < 0.05$. Scale bar = 100 μm.

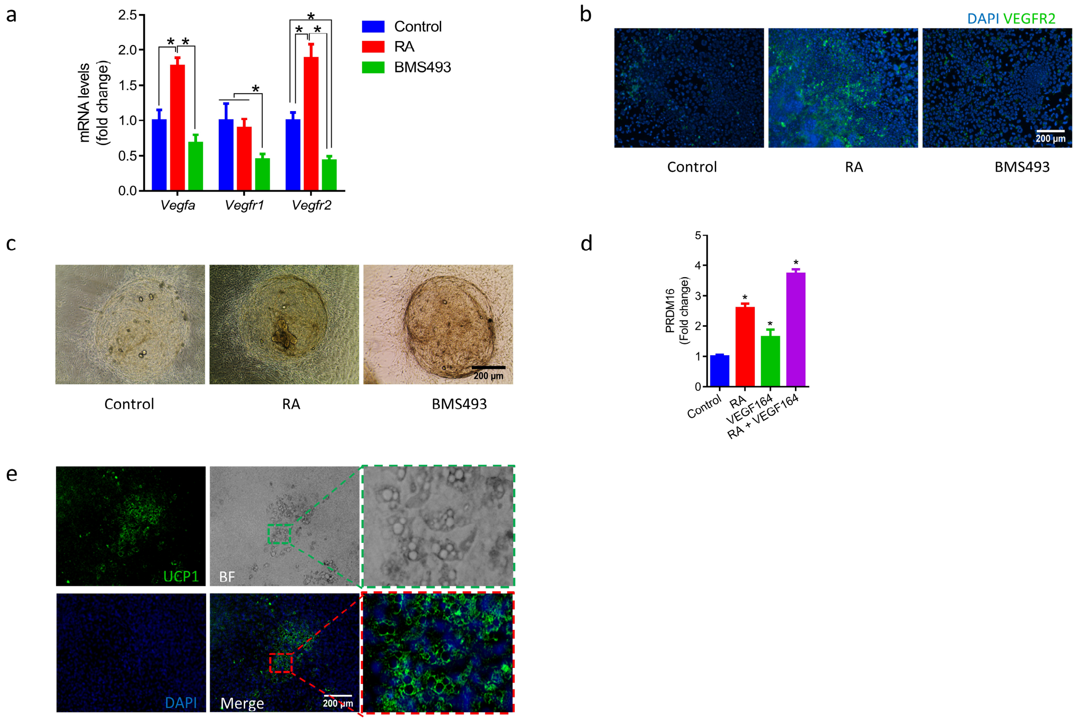

Supplementary Figure S6. RA and VEGF signaling in brown adipogenesis of P19 embryo stem cell line. (a) Expression of angiogenic genes, (b) VEGFR2 positive cells and (c) representative images showing embryo bodies and the expansion of cells after 7 days of adipogenesis. (d) Quantification of PRDM16 content (related to Figure 6c). (e) UCP1 positive cells after 26 days of brown adipogenesis. Data presented are mean ± SEM, n = 6, *$p < 0.05$. Scale bar = 200 µm

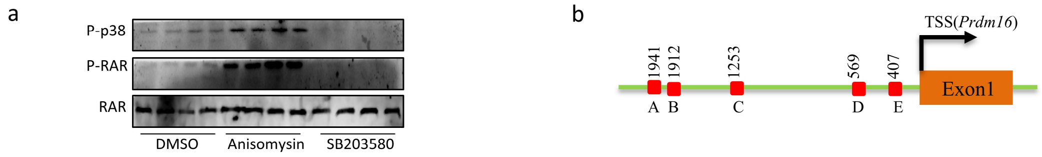

Supplementary Figure S7. RA and VEGF activates p38MAPK to recruit RAR to the *Prdm16* promoter. (a) P-p38 and P-RAR protein content in EBs treated with indicated chemicals for 4 h. (b) Putative retinoic acid response elements (pRARE) on the *Prdm16* promoter.

Supplementary Video S1

Representative video showing iWAT of PDGFRα tracking mice at 24h after tamoxifen injection.

Supplementary Video S2

PDGFRα tracking mice were injected with tamoxifen and, then, injected with vehicle (DMSO) 24h later; video shows iWAT morphology on day 8.

Supplementary Video S3

PDGFRα tracking mice were injected with tamoxifen and, then, injected with RA 24h later; video shows PDGFRα^{+} cells derived adipocytes in iWAT on day 8.
